# In vivo screening of flavonoid compounds revealed quercetin as a potential drug to improve recovery of angiostrongyliasis after albendazole treatment

**DOI:** 10.1371/journal.pntd.0012526

**Published:** 2024-09-30

**Authors:** Ho Yin Pekkle Lam, Yu-Ting Huang, Ting-Ruei Liang, Shih-Yi Peng

**Affiliations:** 1 Department of Biochemistry, School of Medicine, Tzu Chi University, Hualien, Taiwan; 2 Institute of Medical Science, Tzu Chi University, Hualien, Taiwan; 3 Department of Laboratory Medicine and Biotechnology, Tzu Chi University, Hualien, Taiwan; 4 Department of Pathology, Tri-Service General Hospital, National Defense Medical Center, Taipei, Taiwan; 5 PhD Program in Pharmacology and Toxicology, Tzu Chi University, Hualien, Taiwan; University of Agricultural Sciences and Veterinary Medicine Cluj-Napoca, Life Science Institute, ROMANIA

## Abstract

Human angiostrongyliasis, caused by consuming the larva stage of *Angiostrongylus cantonensis*, is an infectious disease involving the central nervous system (CNS) and ophthalmic system. Current treatment of angiostrongyliasis involves albendazole accompanied by analgesics and corticosteroids. However, long-term use of corticosteroids may lead to significant adverse effects. In the current study, we screened through different potentially effective flavonoid compounds and identified quercetin as an effective anti-inflammatory agent in an angiostrongyliasis mouse model. Our results identified that quercetin may reverse the neurological defects in mice with angiostrongyliasis. The brain pathology and inflammatory status were also improved by albendazole-quercetin co-therapy. Further analysis showed that albendazole-quercetin co-therapy had a better therapeutic effect than albendazole or quercetin monotherapy. This therapeutic effect was achieved by inhibiting the brain inflammasome activation and apoptosis. Albendazole-quercetin co-therapy also leads to the inhibition of brain IL-5, possibly leading to improved pathology. Our results here proved that quercetin may serve as a potential adjuvant drug in treating human angiostrongyliasis.

## Author summary

Human infection following accidental consumption of *Angiostrongylus cantonensis* can cause eosinophilic meningoencephalitis. Albendazole is the drug of choice for treatment, followed by continuous use of analgesics and corticosteroids, which may have safety concerns. On the other hand, flavonoids are derived from natural plants and have been shown to improve cognitive function by inhibiting the central nervous system (CNS) inflammation. However, the effect of flavonoids combined with albendazole in treating angiostrongyliasis is still unclear. In the current study, we aimed to investigate the therapeutic potential of flavonoids in combination with albendazole for *A*. *cantonensis* infection. Our results found that quercetin exhibited the most significant therapeutic effect with albendazole, by which the combined regimen improved the brain histopathology and recovered the mice’s neurological functions. This combined therapy also reduces inflammasome activation, pyroptosis, apoptosis, and immune imbalance in the infected mouse brains. Our current study indicated that quercetin possessed the potential as an anti-inflammatory drug and may be helpful in the treatment of *A*. *cantonensis-*induced meningoencephalitis.

## Introduction

Albendazole is currently the drug for angiostrongyliasis, and is supported with analgesics and corticosteroids to achieve a better therapeutic response [1,2]. However, long-term use of corticosteroids can lead to significant adverse effects such as osteoporosis, muscle weakness, and increased risk of infections [3,4]. Therefore, new and effective adjuvant drugs to albendazole are urgently needed.

Traditional Chinese medicine offers new opportunities to tackle infectious diseases as they can be used complementarily or even as alternatives to established therapies [5–7]. These Chinese herbs have been shown to directly or indirectly kill pathogens [8,9] and demonstrate multiple aspects of effects such as anti-inflammation [10], anti-oxidation [11], and anti-fibrosis [12], which resolves injuries caused by the pathogens. Previously, several Chinese herbal compounds such as tanshinone II-A [13] and Schisandrin B [14] have shown significant effects in reducing the pathology and symptoms in experimentally *Angiostrongylus cantonensis*-infected mice, and these drugs showed strong synergism combined with albendazole. However, more research is needed to determine the clinical applications of these compounds. At the same time, more compounds may need to be investigated to find the most efficient one for clinical application.

Flavonoid compounds are abundant in plants and fruits and are commonly consumed in diets. It shows good anti-inflammatory properties, inhibiting many regulatory signalings such as nuclear factor kappa-light-chain-enhancer of activated B cells (NF-κB) signaling, inflammasome activation, gasdermin D (GSDMD)-mediated pyroptosis, and tumor necrosis factor-alpha (TNF-α) signaling [15,16]. Flavonoid compounds have also been investigated in several disease models such as Alzheimer’s disease, Parkinson’s disease, ischemic stroke, and epilepsy, revealing strong beneficial effects against neuroinflammation [17]. Flavonoids are classified into seven subclasses according to their chemical structure: flavan-3-ols, flavones, flavonols, flavanones, anthocyanins, chalcones, and isoflavonoids [18]. In this study, several flavonoid compounds from different subclasses were tested with albendazole to find the most suitable compound to improve the recovery in *A*. *cantonensis*-infected mice. Their inflammatory and neuroprotective potential against angiostrongyliasis were also investigated.

## Materials and methods

### Ethics statement

All experimental protocols involving animals were reviewed and approved by the Institutional Animal Care and Use Committees (IACUC) of Tzu Chi University (No. 104076-A and 109066).

### Animals and parasites

Male BALB/c mice and Sprague-Dawley rats were obtained from the National Laboratory Animal Center, Taipei, Taiwan. Animals were housed in the animal center at Tzu Chi University, and all handling conditions were maintained according to standard practices. *Angiostrongylus cantonensis* was previously isolated from naturally infected Giant African land snails, *Achatina fulica*. The freshwater snail, *Biomphalaria glabrata*, was used as an intermediate host for life cycle establishment.

To perform infection of *A*. *cantonensis* in the mice, infected *B*. *glabrata* was sacrificed and digested with 0.6% pepsin (pH 2) to release third-stage larva (L3). Twenty-five L3 was counted and mixed with sterile water before being introduced to the mice by oral gavage.

### Animal treatment

Two experiments were done in this study. Mice were eight weeks old at the beginning of all experiments.

The first experiment aimed to screen and identify a potential flavonoid compound that can improve the recovery of infected mice after albendazole treatment. After infection, mice were randomly divided into different groups (n = 3 mice per group). At 14 days post-infection, mice were treated with 20 mg/kg/day of albendazole for seven days and 20 mg/kg/day of different flavonoid compounds, including hesperidin, quercetin, naringenin, casticin, wogonin, vitexin, and tanshinone IIA (all compounds were purchased from Chengdu Biopurify Phytochemicals, Chengdu, China), for 14 days.

The second experiment aimed to investigate the therapeutic effect of albendazole-quercetin co-therapy against angiostronyliasis. In this experiment, infected mice were treated with 20 mg/kg/day of albendazole for seven days and/or 10, 20, or 40 mg/kg/day of quercetin for 14 days at 14 days post-infection (n = 5 mice per group).

The body weight of the mice was measured every week. In both experiments, the uninfected and infected control group was fed sterile water under the same procedures. All the mice were sacrificed at day 28 post-infection. Upon sacrifice, the brain was dissected into two hemispheres for histology examination and protein quantification.

### Neurofunctional tests

Mice were subjected to different neurofunctional tests at the beginning of the experiment and every seven days. For the beam walk test, which investigates the mice’s ability to maintain balance, mice were placed at a 1 cm × 70 cm beam, 50 cm above the platform. The time for the mouse to cross the beam was recorded with a 120-second cut-off time. The hindlimb clasping scores were used to detect ataxia and the mice’s ability to control the muscle. Mice were suspended for 10 s, and a score was assigned according to the following criteria: 0, the mice showed normal escape extension; 1, the mice retracted their hindlimb toward the abdomen but not touching the abdomen; 2, the mice retracted their hindlimb and touched the abdomen; 3, the mice showed immobility with both hindlimbs clasped and touched the abdomen. The hot plate test, which investigates the pain response in mice, was done by placing the mice on a 55°C hot plate, and the time for their first paw reaction was recorded with a 15 s cut-off time. The wire hang test was done to detect neuromuscular abnormalities in the mice. The mice were hung upside down on a wire screen for 120 seconds, and the time the mouse fell was recorded. The vertical pole test, which assesses the motor dysfunction of the mice, was done by placing the mice at the top of a 50 cm pole. The time for the mice to turn around and climb down the pole was recorded with a 120-second cut-off time. Time was recorded as 120 seconds if the mice fell from the pole. At least three trials were tested in each experiment, and the average was calculated.

### Histological processing and examination

Brains were fixed by 10% neutral buffered formalin and dehydrated in a series of graded dilutions of alcohols. The tissues were cut into thin sections after immersion in xylene and molten paraffin. Slides were stained with hematoxylin and eosin (H&E) and cresyl violet as described previously [5]. Subsequently, H&E-stained sections were examined and scored for meningitis, hemorrhage, and encephalitis, with each criterion scored as 0, absent; 1, mild; 2, moderate; and 3, pronounced. Cresyl violet-stained sections were counted for dark-stained neurons to estimate apoptotic neurons. Sections were examined blinded and at least ten random fields were examined in each section.

### Western blotting

Extracted protein was resolved on sodium dodecyl sulfate (SDS)-polyacrylamide gel electrophoresis (PAGE) and was transferred to a polyvinylidene difluoride (PVDF) membrane (EMD Millipore, Danvers, MA, USA). After blocking, the membranes were incubated overnight with antibodies against α-tubulin (GeneTex, Irvine, CA, USA), NLRP1B (Santa Cruz Biotechnology, Dallas, TX, USA), NLRC4 (EMD Millipore), caspase-1 (Cell Signaling, Danvers, MA, USA), interleukin-1β (IL-1β; Abclonal, Woburn, MA, USA), interleukin-18 (IL-18; Abclonal), gasdermin D (GSDMD; Santa Cruz Biotechnology), caspase-3 (Cell Signaling), and BCL-2 (GeneTex). Thereafter, membranes were incubated with horseradish peroxidase (HRP)-conjugated mouse or rabbit secondary antibodies (EMD Millipore) for 1 h followed by visualization with enhanced chemiluminescence (ECL) reagents (EMD Millipore). The band intensities of the proteins were quantified by Image J software (v1.46) and were normalized by that of α-tubulin or the uncleaved band.

### RNA extraction and real-time quantitative PCR (RT-qPCR)

Brain samples were homogenized in TRIzol reagent (Invitrogen, Thermo Scientific). RNA was extracted using the standard chloroform extraction method and five micrograms of total RNA were used to synthesize cDNA using a GScript first-strand synthesis kit (Thermo Scientific). RT-qPCR was performed using a 2× qPCRBIO SyGreen Blue Mix Lo-ROX (PCR Biosystems, London, UK) on a Rotor-Gene Q instrument (QIAGEN, Germantown, MD, USA). RT-qPCR conditions included an initial denaturation of 95°C for 2 min, followed by 45 cycles of 95°C for 5 s, 60°C for 10 s, and 72°C for 20 s. The primers used in this study are shown in the S1 Table. Relative gene expression was calculated using the 2^−ΔΔCT^ method with *β-actin* as the housekeeping gene.

### Measurement of cytokine levels

Serum and brain lysate were analyzed for cytokine levels by an enzyme-linked immunosorbent assay (ELISA) kit (Cat#: 432604 for IL-1β; BioLegend, San Diego, CA, USA; Cat#: 88-7711-44 for IL-4, IL-10, IL-2, and IFN-γ; Cat#: 88-7054-22 for IL-5; Thermo Fisher Scientific). Protein concentrations of brain homogenates were determined by the Bradford method using a Bio-Rad Protein Assay Dye (Bio-Rad Laboratories, Hercules, CA, USA).

### Statistical analysis

Data were analyzed by GraphPad Prism 6.01 software (GraphPad Software, San Diego, CA, USA), and presented as mean ±S.D. unless stated otherwise. Statistical comparisons were performed by one-way ANOVA with a Tukey’s honest significant difference (HSD) post-hoc test. A *p*-value less than 0.05 indicates statistical significance.

## Results

### Screening of different flavonoid compounds revealed quercetin as a potential adjuvant drug to be used with albendazole against angiostrongyliasis

**Fig 1:**
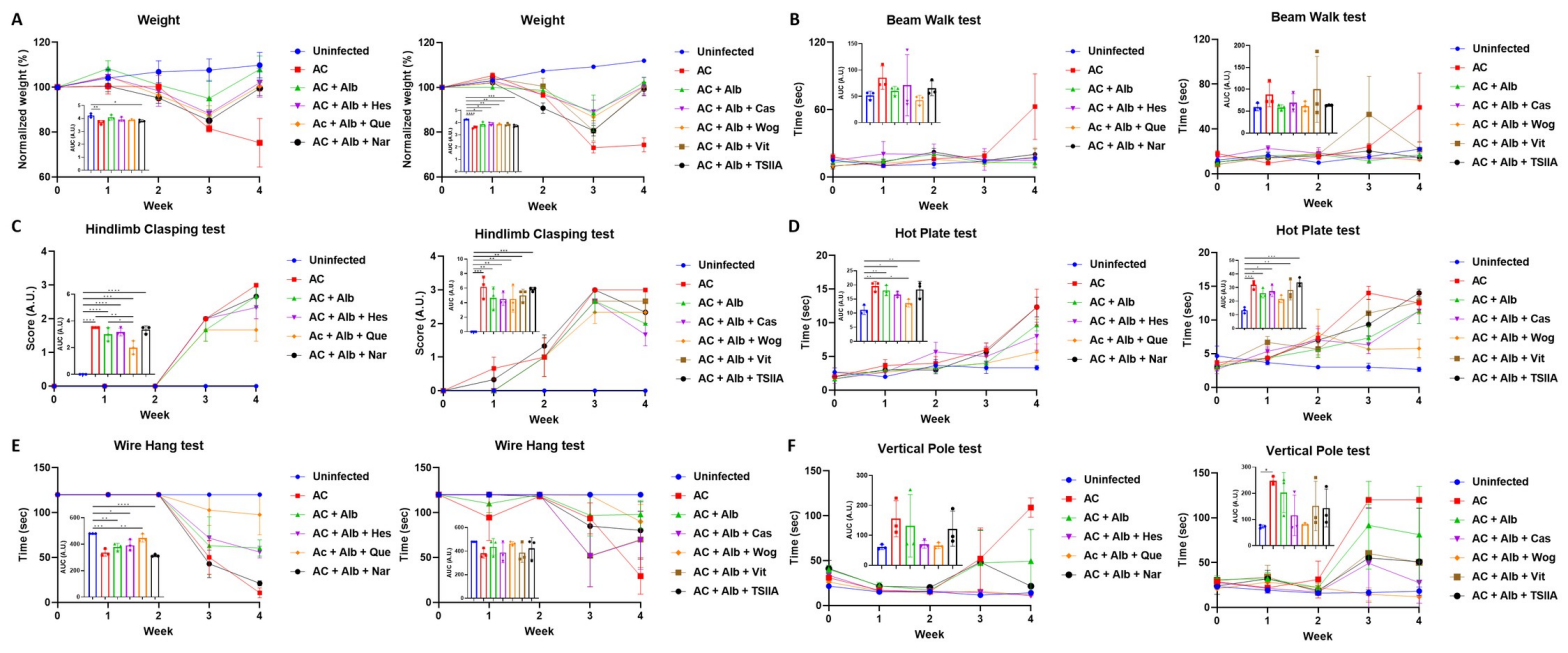
Neurological function tests of mice treated with albendazole and different flavonoid compounds. (A) Normalized body weight of the mice. Weight was normalized to that at the beginning of the experiment. *Angiostrongylus cantonensis*-infected mice (AC) with different treatments were subjected to (B) beam walk test, (C) hindlimb clasping test, (D) hot plate test, (E) wire hang test, and (F) vertical pole test. In each of the subfigures, the left panel shows the results of mice treated with albendazole (Alb) and hesperidin (Hes), quercetin (Que), and naringenin (Nar) whereas the right panel shows the results of mice treated with albendazole and casticin (Cas), wogonin (Wog), vitexin (Vit), and tanshinone IIA (TSIIA). n = 3 mice in each group. Data are presented as mean ± S.E.M., and area under curve (AUC) data are presented as mean ± S.D. * *p* < 0.05; ** *p* < 0.01; *** *p* < 0.001; and **** *p* < 0.0001. Significance determined by one-way ANOVA.

While we observed a significantly decreased body weight in mice with angiostrongyliasis, albendazole monotherapy or albendazole-flavonoids co-therapy slightly recovered the body weight loss. However, the additional use of flavonoid compounds to albendazole did not respond better than the albendazole monotherapy (Fig 1A). The mice’s neurological functions were then analyzed by beam walk test, hindlimb clasping test, hot plate test, wire hang test, and vertical pole test. Mice receiving albendazole monotherapy or combined regimens performed slightly better in the beam walk test than in the infected control group, but not within each treatment group (Fig 1B). On the other hand, the hindlimb clasping test, hot plate test, wire hang test, and vertical pole test suggested that quercetin, among all tested compounds, yielded a better neurological recovery when combined with albendazole (Fig 1C–1F).

**Fig 2:**
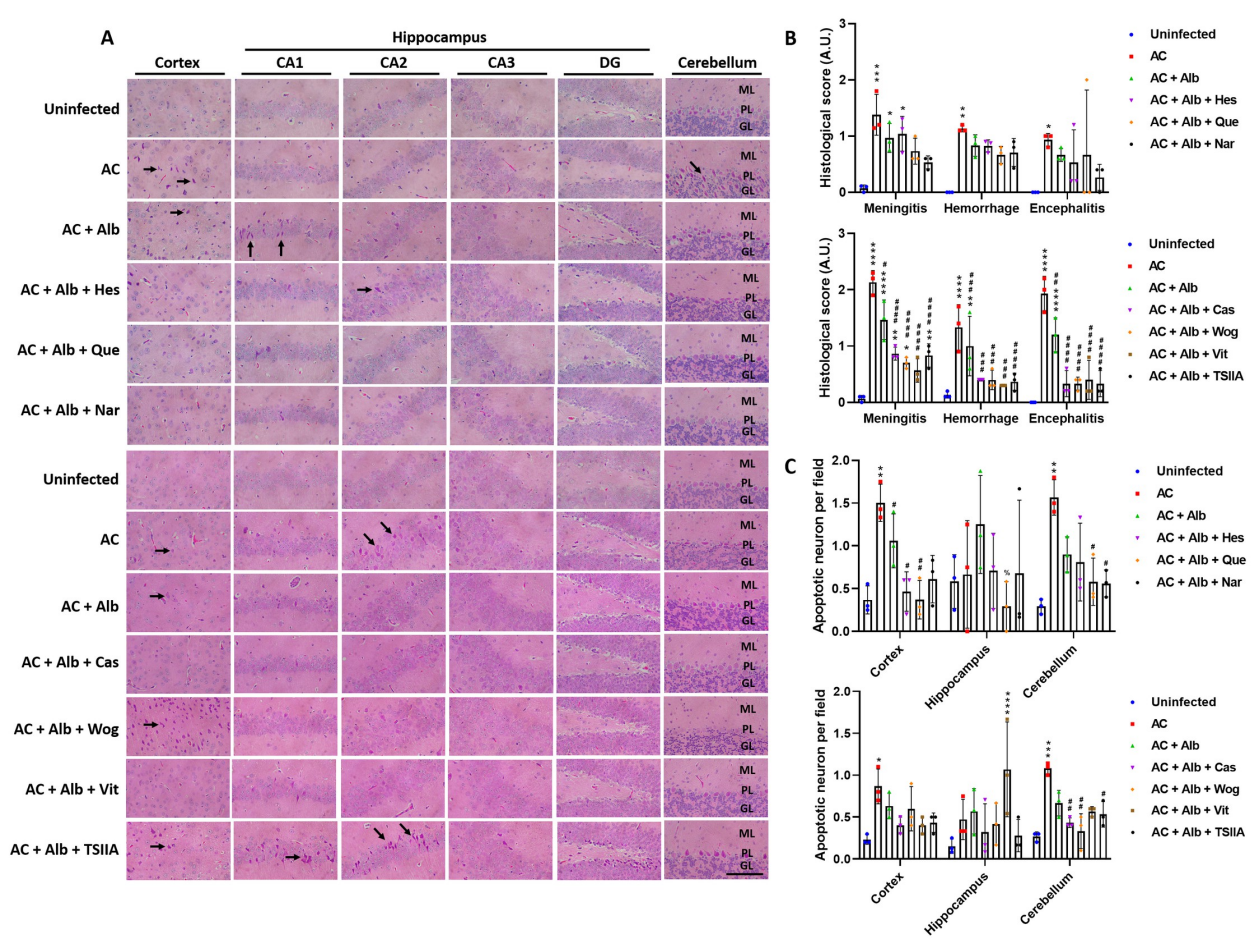
Brain histopathology of mice treated with albendazole and different flavonoid compounds. (A) Representative H&E-stained histological images of the brain (arrows pointing to apoptotic neurons) and corresponding (B) histological score. (C) The number of apoptotic neurons counted in the H&E-stained sections. Scale bars correspond to 100 μm (at 400× magnification). AC, *Angiostrongylus cantonensis*-infected mice; Alb, albendazole; Hes, hesperidin; Que, quercetin; Nar, naringenin; Cas, casticin; Wog, wogonin; Vit, vitexin; TSIIA, tanshinone IIA; CA, Cornu Ammonis; DG, dentate gyrus; ML, molecular layer; PL, Purkinje layer; GL, granular layer. n = 3 mice in each group. Data are presented as mean ± S.D. * *p* < 0.05; ** *p* < 0.01; *** *p* < 0.001; and **** *p* < 0.0001 compared to control group. # *p* < 0.05; ## *p* < 0.01; ### *p* < 0.001; and #### *p* < 0.0001 compared to infected group. Significance determined by one-way ANOVA.

Further analysis revealed that quercetin and naringin, although not statistically significant, improved brain histological damage compared to the infected control group and the albendazole monotherapy group. On the other hand, casticin, wogonin, Vit, and TSIIA-containing regimens showed significantly better histopathological improvement than the infected control group (Fig 2A and 2B). Apoptotic neurons showing acidophilic Nissl substance were also counted, showing fewer apoptotic neurons in the albendazole-quercetin co-therapy group than in the infected control group and the albendazole monotherapy group (Fig 2A and 2C).

**Fig 3:**
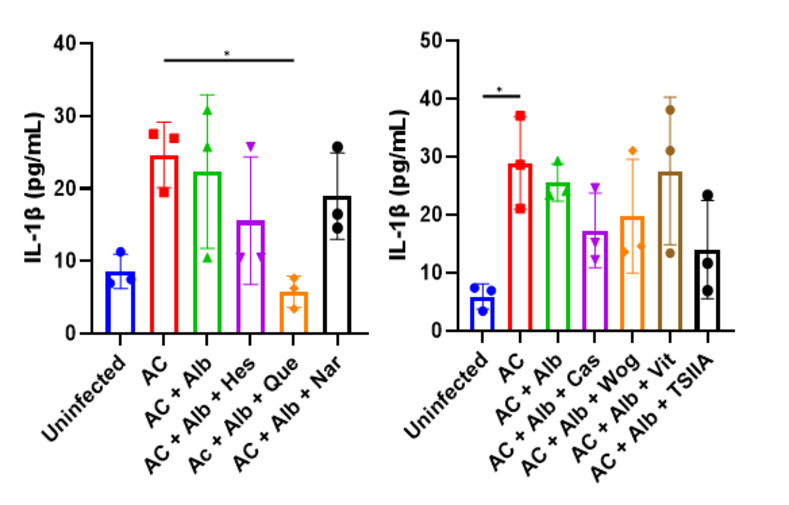
Serum IL-1β levels of mice treated with albendazole and different flavonoid compounds. The left subfigure shows the results of *Angiostrongylus cantonensis*-infected mice (AC) treated with albendazole (Alb) and hesperidin (Hes), quercetin (Que), and naringenin (Nar) whereas the right subfigure shows the results of mice treated with albendazole and casticin (Cas), wogonin (Wog), vitexin (Vit), and tanshinone IIA (TSIIA). n = 3 mice in each group. Data are presented as mean ± S.D. * *p* < 0.05 compared to control group. Significance determined by one-way ANOVA.

Finally, the general status of the mice was investigated by measuring their serum IL-1β levels. The results suggested that only mice receiving albendazole-quercetin co-therapy significantly lowered the inflammatory reaction compared to the infected control (Fig 3), indicating that quercetin might contribute to faster resolution of the inflammation in *A*. *cantonensis*-infected mice.

### Albendazole-quercetin co-therapy improves neurological functions of mice with angiostrongyliasis

**Fig 4:**
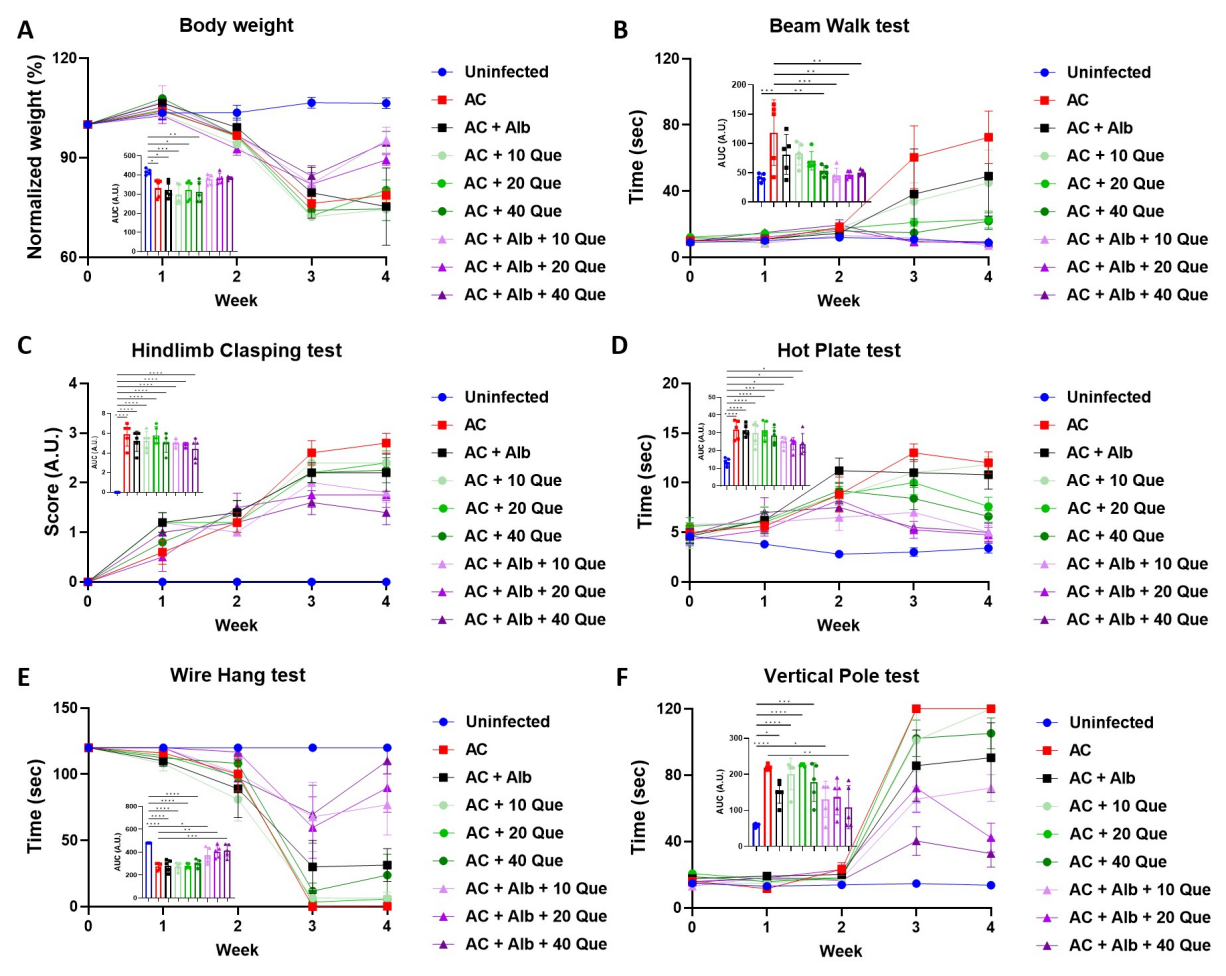
Neurological function tests of mice treated with albendazole and different doses of quercetin. (A) Normalized body weight of the mice. Weight was normalized to that at the beginning of the experiment. *Angiostrongylus cantonensis*-infected mice (AC) treated with albendazole (Alb) and different doses of quercetin (Que) were subjected to (B) beam walk test, (C) hindlimb clasping test, (D) hot plate test, (E) wire hang test, and (F) vertical pole test. n = 5 mice in each group. Data are presented as mean ± S.E.M., and area under curve (AUC) data are presented as mean ± S.D. * *p* < 0.05; ** *p* < 0.01; *** *p* < 0.001; and **** *p* < 0.0001. Significance determined by one-way ANOVA.

The above results have suggested quercetin as a potential compound to use with albendazole in treating angiostrongyliasis. To further investigate the therapeutic effect of quercetin, *A-cantonensis*-infected mice were treated with different concentrations of quercetin, with or without albendazole. Quercetin or albendazole monotherapy did not improve the body weight of infected control mice. On the other hand, albendazole-quercetin co-therapy improved the mice’s body weight, although the effects were not dependent on quercetin concentration (Fig 4A). Similar results can be seen in neurological function tests, showing a better neurological recovery of mice with albendazole-quercetin co-therapy than in albendazole or quercetin monotherapy (Fig 4B–4F).

### Albendazole-quercetin co-therapy dose-dependently suppresses brain inflammation and apoptosis in mice with angiostrongyliasis

**Fig 5:**
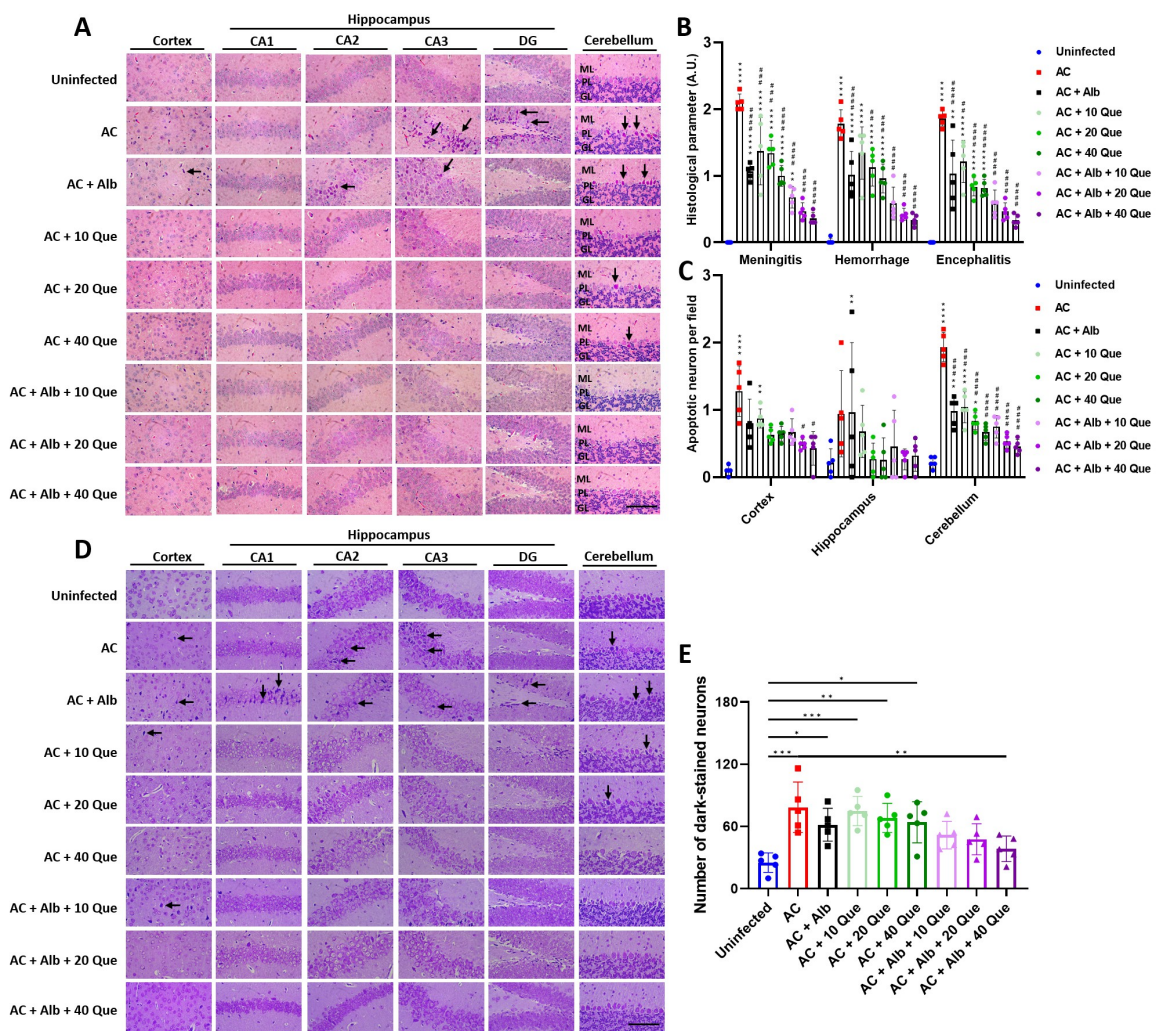
Brain histopathology of mice treated with albendazole and different doses of quercetin. (A) Representative H&E-stained histological images of the brain (arrows pointing to apoptotic neurons), the corresponding (B) histological score, and (C) the number of apoptotic neurons. (D) Representative cresyl violet-stained histological images of the brain (arrows pointing to apoptotic neurons) and the corresponding (E) number of darkly-stained neurons. Scale bars correspond to 100 μm (at 400× magnification). AC, *Angiostrongylus cantonensis*-infected mice; Alb, albendazole; Hes, hesperidin; Que, quercetin; Nar, naringenin; Cas, casticin; Wog, wogonin; Vit, vitexin; TSIIA, tanshinone IIA; CA, Cornu Ammonis; DG, dentate gyrus; ML, molecular layer; PL, Purkinje layer; GL, granular layer. n = 5 mice in each group. Data are presented as mean ± S.D. * *p* < 0.05; ** *p* < 0.01; *** *p* < 0.001; and **** *p* < 0.0001 compared to control group. ## *p* < 0.01; ### *p* < 0.001; and #### *p* < 0.0001 compared to infected group. Significance determined by one-way ANOVA.

Histopathological analysis revealed a significant improvement in brain pathology in mice receiving albendazole-quercetin co-therapy. While the effect was compatible between quercetin mono-treatment and albendazole mono-treatment, a better response was seen in the albendazole-quercetin co-therapy group compared to that in the albendazole or quercetin monotherapy groups (Fig 5A and 5B). Apoptotic neurons were also decreased in different brain parts, with mice receiving albendazole-quercetin co-treatment showing the most significant improvement (Fig 5A and 5C). Cresyl violet staining was done to validate neuronal apoptosis. While albendazole and quercetin mono-treatment have minimal effect against neuronal cell death, albendazole-quercetin co-therapy resulted in better, dose-dependent protection of the neurons (Fig 5D and 5E).

**Fig 6:**
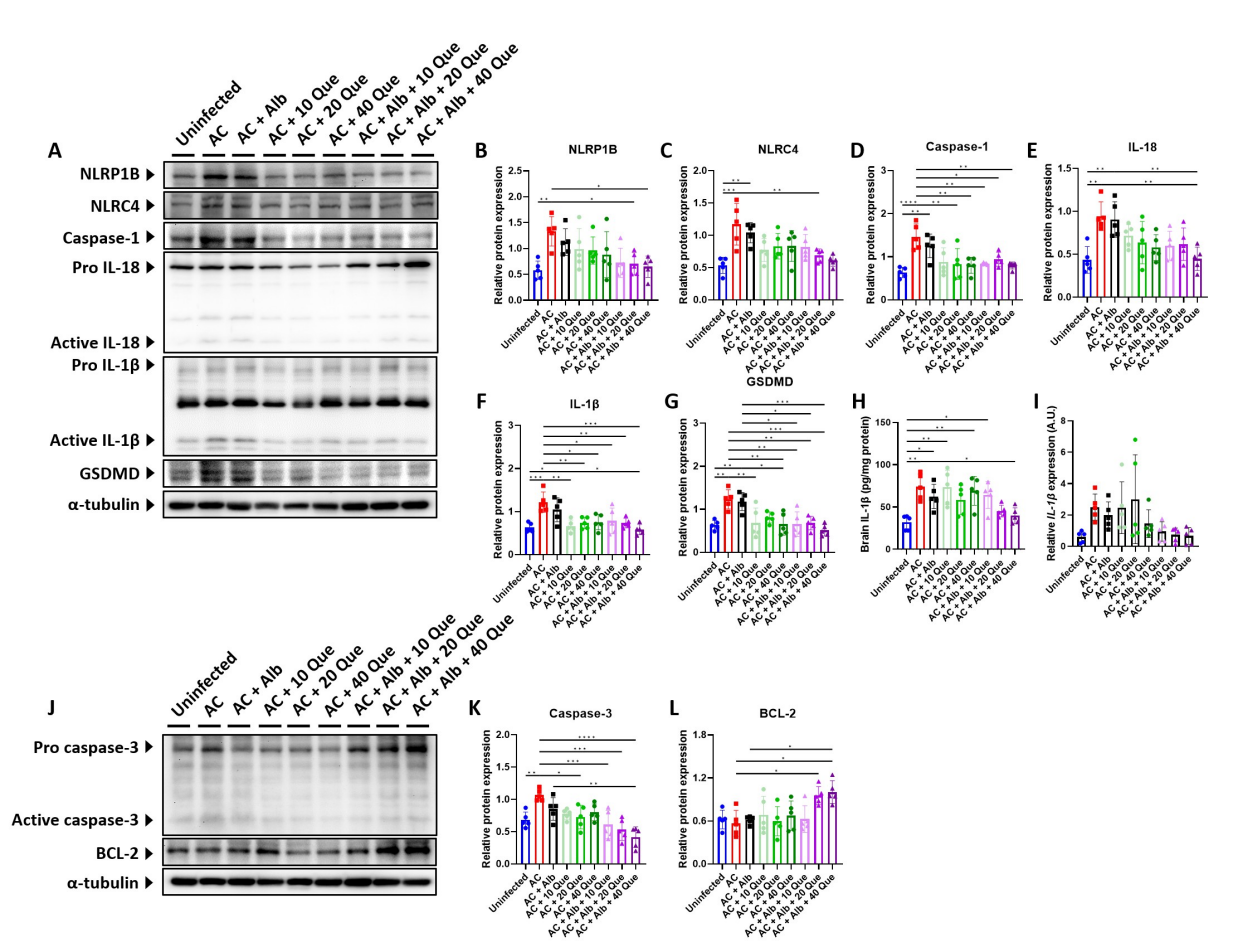
Albendazole-quercetin co-therapy suppresses brain inflammasome activation and apoptosis in mice with angiostrongyliasis. (A) Representative western blot images of inflammasome markers in the brain, and (B-I) their protein expression relative to that of α-tubulin or uncleaved band. (J) Representative western blot images of apoptotic markers in the brain, and (K-L) their protein expression relative to that of α-tubulin or uncleaved band. AC, *Angiostrongylus cantonensis*-infected mice; Alb, albendazole; Que, quercetin. n = 5 mice in each group. Data are presented as mean ± S.D. * *p* < 0.05; ** *p* < 0.01; *** *p* < 0.001; and **** *p* < 0.0001. Significance determined by one-way ANOVA.

To disclose the mechanism of quercetin, activation of NLRP1B and NLRC4 inflammasome pathway was analyzed by western blotting, as *A*. *cantonensis* infection has been shown to activate these pathways to generate an inflammatory response in the brain [19]. The brains of the infected control group showed a significantly increased expression of the inflammasome components. However, albendazole monotherapy did not suppress this activation of inflammatory response. On the other hand, quercetin treatment, with or without albendazole, significantly suppressed the expression of inflammasome components (Fig 6A–6G). By measuring IL-1β concentration and mRNA expression in the brain, a beneficial effect was seen in albendazole-quercetin co-therapy with 20 mg/kg or 40 mg/kg of quercetin (Fig 6H–6I).

Western blotting also confirmed neuronal apoptosis, showing a dose-dependent inhibition of apoptotic caspase-3 and an increase of anti-apoptotic BCL-2 in the albendazole-quercetin co-treated group compared to the infected control group or albendazole monotherapy group (Fig 6J–6L).

### Albendazole-quercetin co-therapy regulates immune status leading to the improvement of pathological outcomes in angiostrongyliasis

**Fig 7:**
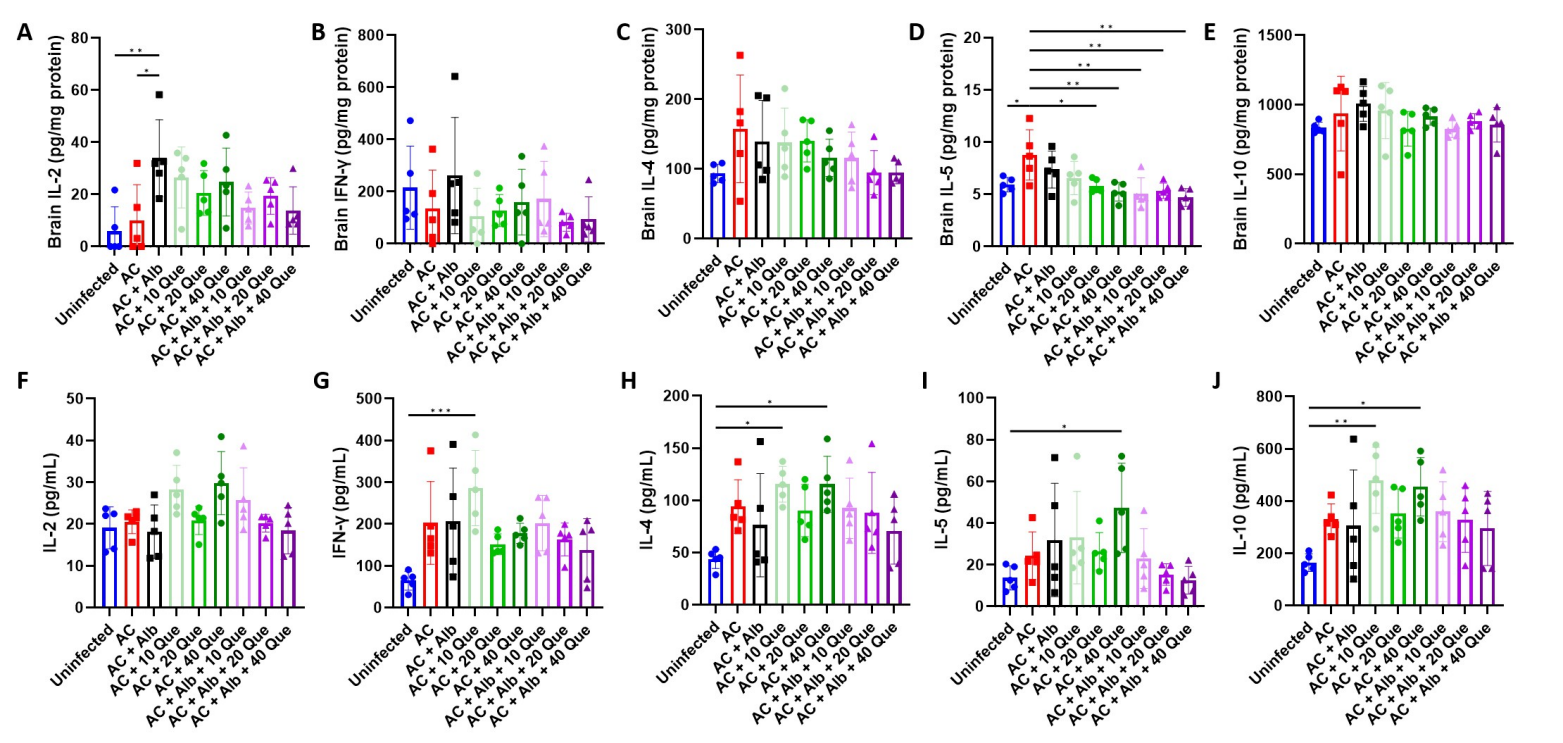
Albendazole-quercetin co-therapy alters cytokine profile in mice with angiostrongyliasis. (A-E) Concentrations of IL-2, IFN-γ, IL-4, IL-5, and IL-10 in the brain. Concentrations were normalized to the total protein concentration of the brain sample. (F-J) Concentrations of IL-2, IFN-γ, IL-4, IL-5, and IL-10 in the serum. AC, *Angiostrongylus cantonensis*-infected mice; Alb, albendazole; Que, quercetin. n = 5 mice in each group. Data are presented as mean ± S.D. * *p* < 0.05; ** *p* < 0.01; and *** *p* < 0.001. Significance determined by one-way ANOVA.

Finally, the immunological changes in the brain were investigated because infiltrating immune cells and their secreted cytokines are one of the major reasons for the *A*. *cantonensis*-induced brain pathology [20,21]. Cytokine concentration measured in the brain revealed an unchanged Th1 cytokine, IL-2, and IFN-γ (Fig 7A and 7B) in the infected control group. With regard to Th2 cytokines, IL-4 and IL-5 (Fig 7C and 7D) levels were increased, but not IL-10 (Fig 7E) in the brains of the infected control group. Albendazole monotherapy did not alter the immune response, but quercetin monotherapy or albendazole-quercetin co-therapy led to a lower IL-5 level (Fig 7D). Cytokine concentrations were also measured in the serum; however, no significant immunological changes were seen in quercetin monotherapy or albendazole-quercetin co-therapy compared to the infected control (Fig 7F–7J). The only seen is that serum IL-5 levels slightly but not significantly decreased in the albendazole-quercetin co-therapy group compared to the infected control group or albendazole monotherapy group (Fig 7I). The differences observed between the brain cytokine level and serum cytokine level may be because the blood only limitedly reflects the immune response in the brain.

## Discussion

For many years, efforts have been made to find alternative anti-inflammatory drugs that can replace the use of steroids with lesser safety concerns [3]. Our current study screened through multiple flavonoids and identified quercetin as a possible drug candidate for treating angiostrongyliasis. Although other compounds also improved the neurological functions of the mice, such as wogonin in the hot plate test or hesperidin in the vertical pole test, the results were not as significant as in quercetin. Therefore, quercetin provided, to some extent, a better recovery of the mice’s neurological functions.

A drug must cross the blood-brain barrier (BBB) to enter the central nervous system for successful neuroprotection. Although flavonoid compounds have been previously shown to provide good neuroprotective effects, studies have pointed out that the ability of flavonoids to cross the BBB depends on their lipophilicity and interactions with efflux transporters [22–24]; by which quercetin has been shown the best biochemical characteristics to penetrate the BBB well [24].

Our experiment found that mice receiving quercetin monotherapy resulted in improved histopathology compared to the infected control group, and quercetin achieves an even more powerful effect when combined with albendazole, as seen in the improved neurological functions (Fig 4) and histopathology (Fig 5). Further investigation revealed that albendazole-quercetin co-therapy hinders inflammasome activation and GSDMD-mediated pyroptosis (Fig 6A–6I). Once the parasite reaches the brain, the pathogen-associated molecular patterns (PAMPs) are recognized by the pattern recognition receptors (PRRs) expressed on the microglia, which then induce activation of NLRP1B and NLRC4 inflammasome [19,25]. Quercetin has been shown to inhibit the phosphorylation of JNK2 and p38 MAPK signaling, leading to unsuccessful inflammasome assembly [26]. In another study, quercetin inhibited the NLRP3 and AIM2 inflammasome by inhibiting the ASC-speck formation [27]. While NLRP1B and NLRC4 can bypass the use of ASC to activate caspase-1 [19,28], another mechanism may be involved in how quercetin inhibits inflammasome activation, which would require future study. Since inflammasome activation was inhibited, suppression of GSDMD-mediated pyroptosis was also reasonable. Albendazole-quercetin co-therapy also inhibited apoptosis, but it did not work well when quercetin was used alone (Fig 6J–6L). This result was similar to the results of the Nissl-stained sections (Fig 5D and 5E). When combined with albendazole, quercetin showed a decreased expression of apoptotic caspase-3 and an increased expression of anti-apoptotic BCL-2 protein. Quercetin has been previously showed to process a strong anti-tumor activity, leading to tumor cell apoptosis [29–31]. Compared to our study, these contradictory results were explained by the tumor-specificity of flavonoids, which showed higher cytotoxicity against tumor cell lines than normal cells [32]. Quercetin has also been shown to attenuate liver cell apoptosis by inhibiting high mobility group box-1 (HMGB1) [33], suggesting a diverse function of quercetin.

Finally, the immune-modulatory effect of quercetin was investigated. However, quercetin treatment, used with or without albendazole, only suppresses IL-5 levels in the brain (Fig 7D). Interleukin-5 mediates parasite-associated host defense, generating eosinophils for pathogen clearance [34]. Therefore, the suppression of brain IL-5 levels may suggest a lesser eosinophils recruitment and, thereby, lesser inflammation, corroborating what we have seen in the histology (Fig 5). Quercetin has been shown to upregulate IFN-γ and downregulate IL-4 expression in normal peripheral blood mononuclear cells [35]. Although this effect was not seen in our study, we cannot exclude the possibility that quercetin may also affect the immune cell subsets in the brain, and future studies investigating each of the single cells in the brain may shed light on this hypothesis.

Quercetin is widely found in fruits and vegetables worldwide [36]. The consumption of these quercetin-containing foods has been shown to be of pharmacological importance. Oral quercetin consumption is absorbed in the small intestine in glycoside and aglycone forms [37], where they can easily diffuse across the BBB and accumulate in the brain as quercetin-3-O-β-d-glucuronide [38], leading to persistent anti-inflammatory effects. An in vivo study suggested that quercetin consumption of up to 25 mg/kg yielded no adverse effects and increased brain mitochondrial biogenesis [39]. Human consumption of up to 2000 mg/day quercetin is also safe [40,41]. Therefore, quercetin may serve as a potential anti-inflammatory drug to use with antihelminthics for angiostrongyliasis.

## Supporting information

S1 Table Primer pairs used in this study. (XLSX)

S1 Data All relevant dataset in this study. (XLSX)

S1 Fig Original blots for Fig 6A and 6J. (TIF)
