# Supplementary figures and images for: In vivo screening of flavonoid compounds revealed quercetin as a potential drug to improve recovery of angiostrongyliasis after albendazole treatment
Source: PLoS Negl Trop Dis. 2024 Sep 30;18(9):e0012526. doi: 10.1371/journal.pntd.0012526 (PMC11476796; doi:10.1371/journal.pntd.0012526)

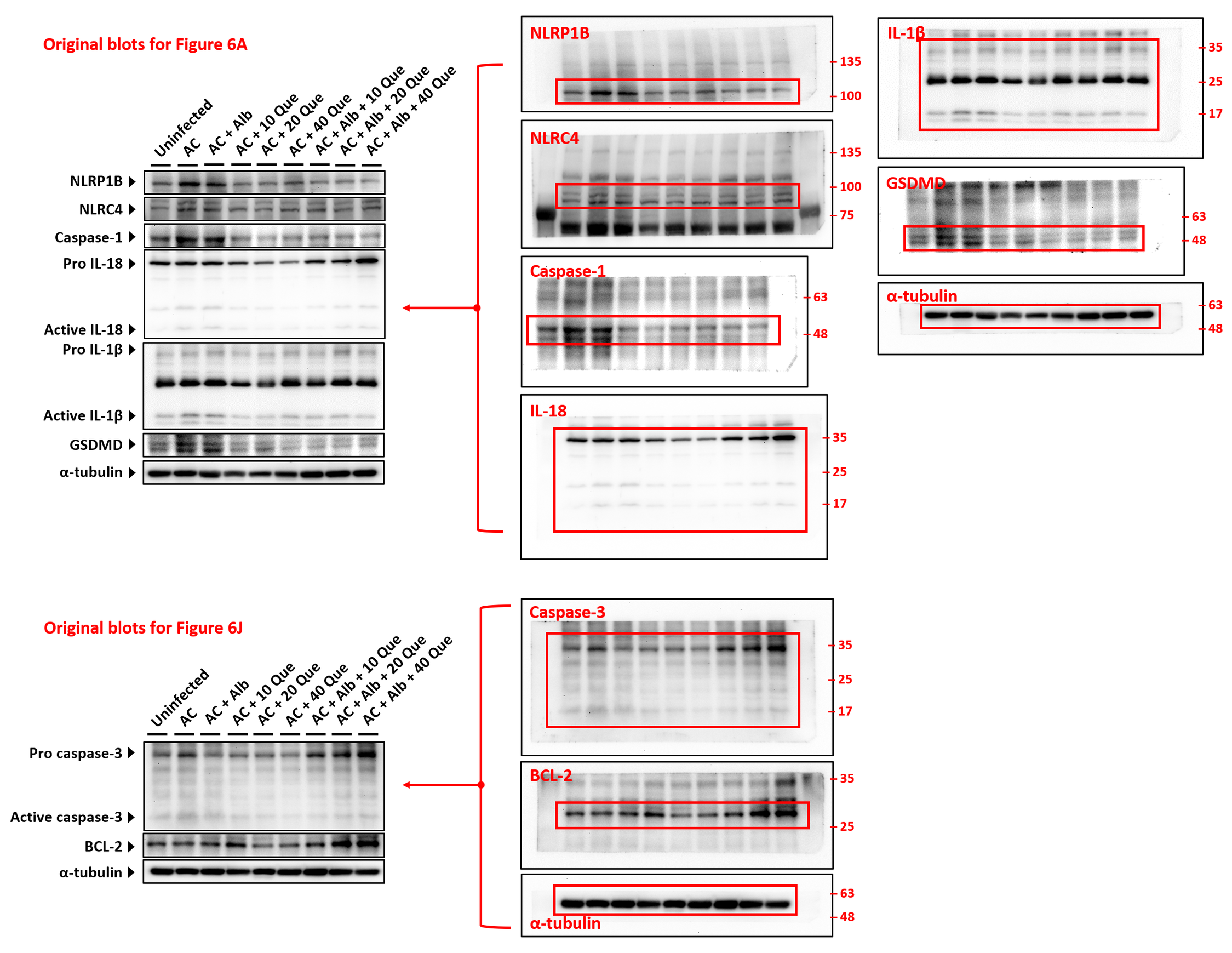

Supplement: S1 Fig — (TIF) [file pntd.0012526.s003.tif]
